# Research on grape leaf disease recognition method based on improved YOLOv8n model

**DOI:** 10.3389/fpls.2025.1695686

**Published:** 2026-02-12

**Authors:** Huiping Guo, Jiarui Cao, Yi Wang, Linrui Rong, Fuzeng Yang, Liangliang Zhu, Weiguo Zhang

**Affiliations:** 1 College of Mechanical and Electronic Engineering, Northwest A&F University, Yangling, China; 2 College of Mechanical and Electronic Engineering, Shaanxi A&F Technology University, Yangling, China

**Keywords:** grape leaves, disease recognition, improved YOLOv8n, variable spraying, deep learning

## Abstract

Grape leaf disease recognition models face challenges such as large model sizes and a lack of classification for various disease types. This study proposes an enhanced grape leaf disease recognition model using an improved version of YOLOv8n, addressing these limitations. To improve performance, several modifications were made to the original YOLOv8n architecture. First, the G-bneck module was introduced into the backbone network to replace the ConvModule, enhancing feature extraction. Simultaneously, the simSPPF module was adopted to replace the SPPF, improving computation speed while preserving feature extraction capabilities. Next, the UIB module was incorporated into both the backbone and neck networks, replacing the Bottleneck module in C2f. This modification resulted in the C2F-UIB module, which reduced parameter size and computational load by eliminating the skip connection. Additionally, the LInner-CIoU loss function was introduced to replace the traditional LCIoU loss in the head network. To accelerate inference and handle irregular, missing, or occluded images, partial convolution and convolution parameter sharing were integrated into the detection head.Experimental results demonstrated that the proposed model outperforms other models, including YOLOv3-tiny, YOLOv5n, and YOLOv6n, in terms of average accuracy. The improved YOLOv8n model achieved an accuracy of 97.3%, with a model size of 3.53MB and a processing speed of 228.55 frames per second (FPS). When deployed to a spraying device, the model maintained an average accuracy of 89.3% and an average processing time of 5.18 seconds. This study successfully addresses the challenges of grape leaf disease recognition by improving model accuracy, size, and inference speed. The proposed model enables accurate and rapid identification of grape leaf diseases in natural environments, offering significant potential for precision agriculture, particularly in the development of effective grape disease management and control technologies.

## 1 Introduction

Grapes are one of the most important fruit crops globally, not only forming the core of the fruit, wine, and dried fruit industries but also contributing to agricultural economy development and regional ecological balance. By 2023, grape planting in China covered approximately 756,000 hectares, making it a major producer worldwide (Liu et al., 2024). The yield and quality of grapes are easily affected by diseases. Common grape leaf diseases include *Erysiphe necator*, *Grapevine mosaic virus*, *Plasmopara viticola*, and *Phomopsis viticola* (Chu et al., 2023). Traditional grape leaf disease identification mainly relies on manual experience, which is susceptible to the influence of experience and disease complexity, resulting in low identification accuracy and being unfavorable for orchard management (Javidan et al., 2024). Therefore, the automatic and accurate identification of grape leaf diseases is crucial for the precise pesticide application and refined management of orchards.

In the early stage, traditional image processing methods were used for disease identification. Yousef et al. (2022) conducted apple leaf disease identification based on digital image processing and a sparse coding model, achieving an average accuracy of 85%; Koushik et al. (2018) used a genetic algorithm and support vector machine for soybean disease identification, with an accuracy of 90.91%; Hayit et al. (2023) applied a KNN classifier based on color and texture features to identify chickpea diseases, reaching an accuracy of 94.5%. Although traditional methods have achieved good identification results, they require manual labeling of disease features, demand large datasets, and have large model computation and volume.

There have been numerous research achievements in crop disease identification based on Convolutional Neural Networks (CNNs) (Yang et al., 2025). Lin et al. (2022)proposed a lightweight grape leaf disease identification model called GrapeNet, with a recognition rate of 86.29%; Peng et al. (2022) proposed a grape leaf disease identification algorithm based on an improved MobileNetV2 model, achieving an accuracy of 89.16%; Sagar et al. (2025) put forward a grape leaf disease identification algorithm based on an improved ResNet model, with an accuracy of 94%; Fan et al. (2021) proposed a grape leaf disease identification model based on an improved VGG16, reaching an accuracy of 97.1%; Wang et al. (2021) presented a grape leaf disease identification model based on an improved ECA-SNet, with an accuracy of 98.86%. Under natural conditions, crop diseases are often affected by factors such as light, occlusion, and background, leading to a decrease in identification accuracy. Hence, it is necessary to improve CNN models to enhance their identification performance in complex scenarios. Li and Zhao (2025) proposed a tea leaf disease identification model based on an improved ResNet, with a recognition rate of 93.06%; Hu et al. (2024) put forward a corn disease identification model based on an improved LFMNet, achieving a recognition rate of 94.12%; Xu et al. (2020) proposed a corn disease identification model based on an improved VGG-16, with a recognition rate of 95.33%. Although the above models have high accuracy, they only target a single disease, do not classify diseases by severity, and have not undergone field tests. Therefore, to improve the recognition rate in field tests and deploy the model on mobile devices, this paper proposes a grape leaf disease identification model based on YOLOv8n.

In this study, a self-made dataset was used to train and test the improved model. After deploying the trained model on mobile devices, greenhouse tests were conducted to verify the effectiveness of the proposed grape leaf disease identification model in complex environments. The main contributions of this paper are as follows:

1. Introduce G-bneck to replace ConvModule in the backbone network of YOLOv8n, and use simSPPF to replace the model’s SPPF, so as to improve the computation speed while maintaining the feature extraction capability;
2. Introduce UIB to replace the Bottleneck module in C2f in both the backbone network and the neck network, and remove the skip connection to obtain the C2f-UIB module, thereby reducing the parameter quantity and computation amount of the C2f module;
3. Introduce LInner-CIoU to replace the loss function LCIoU in the head network, and at the same time, introduce partial convolution and convolution parameter sharing mechanism in the detection head to accelerate the inference speed and effectively process images with irregular missing parts or occlusions;
4. Conduct a comparative analysis of several algorithms. The average precision value of the grape disease identification model based on YOLOv8n is higher than that of 4 models including YOLOv3-tiny, YOLOv5n, and YOLOv6n, with a precision rate of 91.2% and a volume of 5.93MB.

The structure of this paper is as follows: Section 2 summarizes the related work; Section 3 introduces the composition of the dataset; Section 4 describes the proposed grape leaf disease identification model based on YOLOv8n, the test environment and training parameter configuration, and the evaluation indicators; Section 5 presents and analyzes the comparative test results of different algorithms, ablation tests, and greenhouse tests; Section 6 is the discussion part; Section 7 provides conclusions and suggestions for future research.

## 2 Related work

There have been good research achievements in the identification of single disease characteristics, but in agricultural production, the disease characteristics of crops vary. Some scholars have improved the traditional CNN recognition model to adapt to the recognition of various diseases. Wang et al. (2022) proposed a grape leaf disease identification model based on improved LMA-CNNs, which identified 4 types of diseases with an average recognition rate of 88.08%. Tariq et al. (2024) proposed a corn leaf disease identification method based on the improved VGG16 model, achieving high-precision identification of multiple diseases such as corn rust, leaf spot disease, and gray spot disease, with an average recognition rate of 94.67%. Swaminathan and Vairavasundaram (2024) proposed an improved rice leaf recognition model based on cnn, with an average recognition accuracy rate of 96.45%. These improved CNN algorithms have further enhanced their ability to extract disease features and have a relatively high accuracy rate in identifying different types of diseases. Roy and Kukreja (2025) proposed a method for rice leaf disease detection and severity estimation based on Vision Transformer (ViT), which classified ten diseases into three levels and conducted feature recognition on a total of 31 features. The highest recognition rate reached 81.7%. Experiments proved that it was significantly superior to the single-task CNN method in severity determination. And it has improved the accuracy of identifying the severity of diseases; Guo et al. (2024) proposed an apple defoliation disease identification model based on improved MobileNetV3 (ET3-MobileNetV3), which categorized 4 types of diseases into 2 severity levels (covering a total of 9 features) and achieved an average accuracy of 95.62%, the severity of leaf diseases has been classified and identified, improving the accuracy of the model. The above-mentioned disease identification models all have high recognition accuracy. When deployed on field operation equipment, in addition to the recognition accuracy, the positioning accuracy in complex backgrounds is also very important. Object detection algorithms such as SSD, YOLO, Faster R-CNN, EfficienDet and Transformer can effectively solve these problems. Sun et al. (2021) proposed an apple leaf disease recognition model based on the SSD algorithm, with a recognition rate of 83.12% and a detection speed of 12.53fps. The on-site detection speed is relatively fast, but the recognition accuracy is insufficient, especially for the detection of small or dense disease spots. Iqbal et al. (2022) constructed an apple leaf disease recognition model through the EfficienDet, YOLOV4 and Faster-RCNN algorithms. On the basis of maintaining a relatively good detection accuracy rate, YOLOV4 has the fastest model running speed, with an accuracy rate of approximately 88% and a detection speed of approximately 42%. Liao et al. (2024) proposed a rice disease identification model based on YOLOV5, with an identification accuracy rate of 94.2%. The YOLO algorithm features lightweight characteristics. The model can maintain a high recognition rate, but its robustness is relatively weak under extreme lighting or occlusion conditions. Nigam et al. (2024) developed a wheat field disease identification model based on the EfficientNet architecture, achieving an identification accuracy rate of 96.68%. The EfficientNet framework introduces an attention mechanism, which can capture fine-grained features of disease spots, improve identification accuracy, and enhance the robustness of the model. Bari et al. (2021) proposed a real-time diagnosis method for rice leaf diseases based on the deep learning Faster R-CNN framework, using the regional candidate network (RPN) to achieve automatic localization and classification of the lesion area. The recognition accuracy rate of this model on multiple common rice disease datasets reached 98.57%, demonstrating strong real-time detection capabilities and generalization performance, effectively enhancing the efficiency and accuracy of disease recognition.

The improved object detection model has higher recognition accuracy in complex environments, and its computing power has been further enhanced, making it more suitable for field deployment. Sun et al. (2022) proposed an improved YOLOv5s apple disease identification model, with an average accuracy of 91.6% and a model size of 2.06MB. Chakrabarty et al. (2024) developed a rice disease recognition model based on an improved Transformer, achieving an accuracy rate of 97%. Mao et al. (2022) proposed a wheat disease recognition model based on the improved Faster R-CNN, with an accuracy rate of 93.56%. While maintaining the detection speed, it significantly improved the accuracy of lesion location and the ability of boundary recognition. He et al. (2022) proposed a corn leaf disease recognition model based on the improved Faster R-CNN, with an accuracy rate of 97.23%. The improved Faster R-CNN has a strong detection ability for fine lesions, but the model structure is complex, the training time is long, and the computational load is large. Ishsk, (2024) proposes a corn leaf disease model based on vision Transformer, with an accuracy rate of 99.24%, but the model structure is complex, the training time is long, and the demand for computing resources is large. Tonmoy et al. (2025) proposed a mobile-friendly hybrid vision Transformer (ViT) plant disease image segmentation model named MobilePlantViT, achieving a recognition accuracy rate of up to 99.55% on multiple plant disease datasets, significantly outperforming traditional lightweight models. However, the robustness of this model in complex natural scenarios still needs to be improved, and its recognition effect on some small sample disease categories is insufficient.

The object detection model has achieved certain results in the research of crop diseases. However, there are still few studies on the classification detection and location recognition of grape leaf diseases, especially in the aspect of mobile deployment. Liu et al. (2024) proposed a detection method for grape leaf diseases based on Fusion Transformer YOLO (FTR-YOLO), which can identify four diseases. The model introduces a lightweight and high-performance VoVNet network and adopts an improved dual-stream PAN+FPN. 2D position embedding and single-scale Transformer encoder were introduced in a feature mAP to simultaneously enhance model performance and detection accuracy. The average precision (mAP) reached 90.67%, the frame rate (FPS) was 44 frames per second, and the parameter count was only 24.5M. Zhang et al. (2024) proposed a disease spot detection method for grape black rot and black measles based on the combination of an adaptive discriminator-enhanced style generative adversarial network and an improved YOLO v7. The MSRCP algorithm was adopted for image enhancement. In the YOLO v7 network framework, the BiFormer attention mechanism was embedded in the feature extraction network, and BiFPN was used instead of PA-FPN, etc. A disease recognition model for grape leaves was constructed, and the accuracy rate of disease spot detection in the model reached 94.1%. Zhang et al. (2024) proposed a disease recognition model for grapes based on the improved YOLOv8 model for six diseases including black rot, leaf blight and downy mildew. By introducing the GhostNetV2 backbone feature extraction network, embedding SPPFCSPC pyramid pooling, and adding the GAM Attention mechanism and other methods to optimize the YOLOv8 algorithm, the accuracy rate of the optimized grape leaf disease recognition model is 97.1%, and the model volume is 50MB. The model detection accuracy is relatively high, but it is large in size, which is not conducive to field deployment. All these methods can automatically identify grape diseases, improve detection efficiency and reduce labor costs. However, these methods for detecting grape diseases are difficult to balance model lightweighting and accuracy, and at the same time lack the function of grading the severity of diseases.

To address the above issues, this study takes grape leaves with diseases and healthy grape leaves as the research objects, and proposes an improved YOLOv8n-based grape leaf disease identification model. The model aims to improve disease identification accuracy, reduce model size, and enable disease severity classification. The grape disease identification model is deployed on an orchard variable spray device, and field verification of its disease identification performance is conducted through orchard pesticide spraying tests.

## 3 Methodology

### 3.1 Image acquisition of the dataset

#### 3.1.1 Data collection

The grapes are grown in facility greenhouses, which are 60m×15m in size. The age of the grapevines is approximately three to five years, with a row spacing of two meters and a plant spacing of one meter. There are many types of grape leaf diseases. This paper selects 5 types of leaves, namely those affected by *Erysiphe necator, Grapevine mosaic virus, Plasmopara viticola, Phomopsis viticola*, and healthy leaves, as the research objects.

The dataset required for the experiment was collected under different light conditions on cloudy and sunny days. The leaves of different grape varieties such as Hutai 8 and Summer Black were photographed at the Grape Planting Experimental Station of Northwest A&F University, Rougu Farm in Yangling District, and Huxian Beautiful Countryside Planting Professional Cooperative. A Intel RealSence D435 camera was used as the shooting device with a resolution of 1920×1080. The shooting time was from March to May and June to August 2023. Based on the principles of labelability of lesion areas, integrity of disease characteristics, and standardization of image quality in the image data, a total of 7383 images were selected from the collected image dataset, including 1673 images of *Erysiphe necator* 1632 images of *Grapevine mosaic virus*, 1613 images of *Plasmopara viticola*, 1642 images of *Phomopsis viticola*, and 823 images of healthy leaves.

#### 3.1.2 Data grade classification

According to the proportion of the disease spot area in the total area of grape leaves and in accordance with the relevant standard 《 GB/T 17980–2004 Pesticides - Guidelines for Field Efficacy Trials (II) 》, the disease infection degree is divided as shown in **Table 1**.

**Table 1:** Classification of grape leaf disease severity.

Types of diseases	Grade of infection	Quantity/amplitude
Erysiphe necator	AI	843
	AII	830
Grapevine mosaic virus	BI	828
	BII	804
Plasmopara viticola	CI	793
	CII	820
Phomopsis viticola	DI	771
	DII	871
Healthy Leaf	H	823

### 3.2 Dataset construction

To enhance the target detection algorithm’s memory of incorrect labels, increase sample robustness, and improve the network’s generalization ability (Zhang et al., 2022 & Li et al., 2022), geometric transformations such as rotation, horizontal flipping, and vertical flipping were applied to the original images. These transformations increase the diversity of disease region positions, thereby reducing the impact of disease region locations on network recognition. In this study, color transformations such as adjusting image saturation and brightness were also performed to mitigate the influence of complex lighting conditions in actual scenarios on the network’s disease detection, thus achieving data augmentation (Bao et al., 2024 & Peng and Li, 2023). By mixing the newly generated disease data with the original data, a total of 73,830 grape leaf disease images were obtained. Considering the demand for optimizing training efficiency, in the data preprocessing stage of this study, the pixel resolution of all input images was standardized. The 1920×1080 pixels were adjusted to 640×640 to enhance the data loading speed and ensure the consistency of the input data.

To reduce overfitting caused by an excessively large training set and improve the accuracy of model training, the image set was divided into a training set and a test set in an 85:15 ratio, ultimately completing the dataset construction. The number of samples for each disease in the training set and test set is shown in **Table 2**.

**Table 2:** Expand the dataset.

Types of diseases	Grade of infection	Training set Quantity/amplitude	Test set Quantity/amplitude	Total	Test set of proportion
Erysiphe necatorI	AI	7168	1262	8430	0.1497
Erysiphe necatorII	AII	7055	1245	8300	0.1500
Grapevine mosaic virusI	BI	7040	1240	8280	0.1497
Grapevine mosaic virusII	BII	6834	1206	8040	0.1500
Plasmopara viticolaI	CI	6740	1190	7930	0.1501
Plasmopara viticolaII	CII	6970	1230	8200	0.1500
Phomopsis viticolaI	DI	6654	1156	7710	0.1499
Phomopsis viticolaII	DII	7404	1306	8710	0.1499
Healthy Leaf	H	6995	1235	8230	0.1501
Total		62755	11075	73830	-

## 4 Model improvement

**Figure 1:**
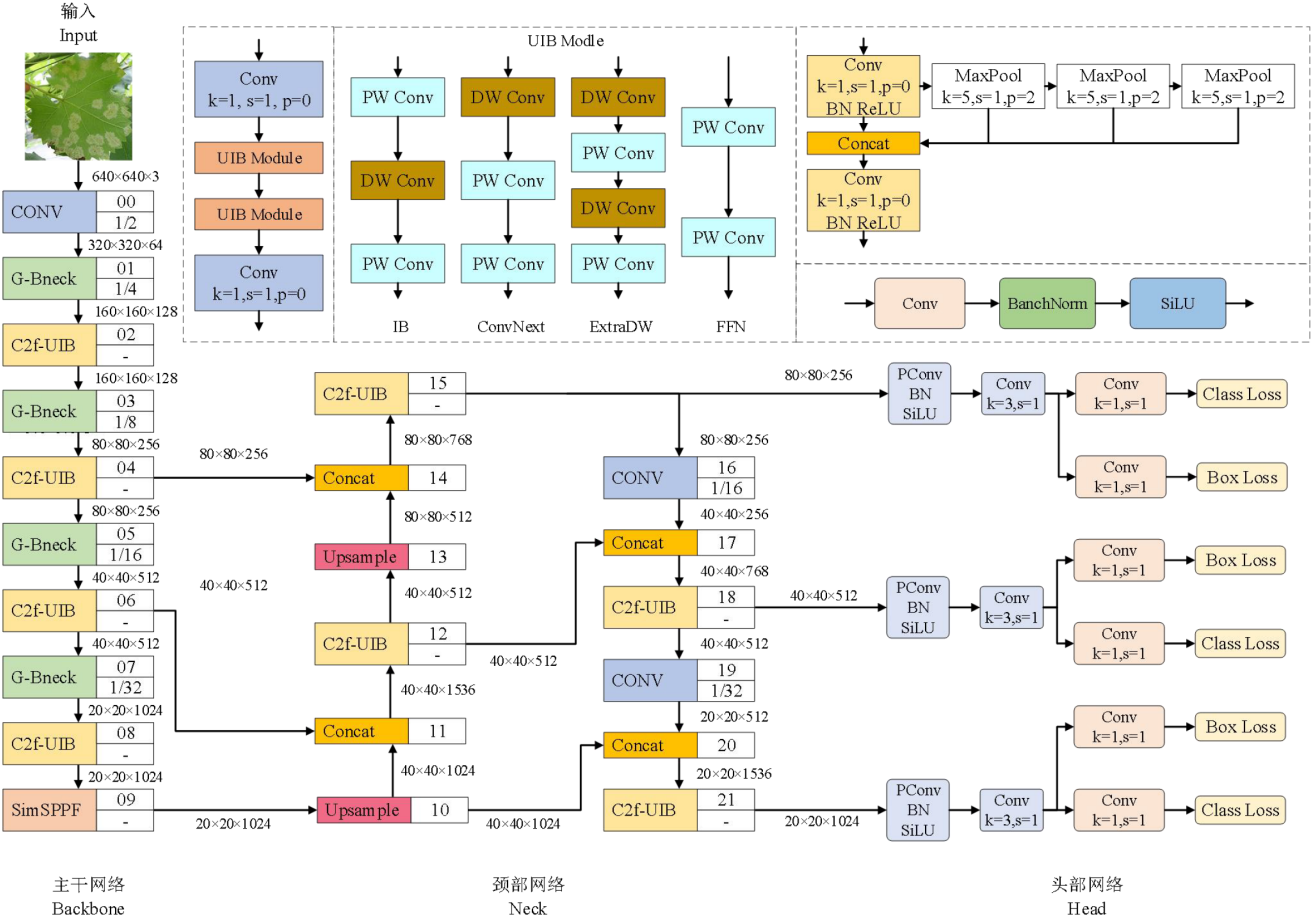
Improved YOLOv8n network structure diagram.

To improve the speed and accuracy of grape leaf disease identification in natural environments, the YOLOv8n model is optimized and improved. (1) In the backbone network, G-bneck is introduced to replace ConvModule, which improves the computing speed while maintaining the feature extraction capability; simSPPF is used to replace the model’s SPPF, which also enhances the computing speed while preserving the feature extraction capability. (2) In the backbone network and neck network, UIB is introduced to replace the Bottleneck in the C2f module, and the C2f-UIB module is obtained after removing the skip connections, thereby reducing the number of parameters and the computation load of the C2f module. (3) In the head network, LInner-CIoU is introduced to replace the loss function LCIoU; meanwhile, partial convolution (PConv) and convolution parameter sharing mechanism are introduced into the detection head, which can accelerate the inference speed and effectively process images with irregular missing parts or occlusions. The structure of the improved network model is shown in **Figure 1**.

### 4.1 YOLOv8n model improvement

#### 4.1.1 Introduce G-bneck to replace ConvModule in the backbone network

**Figure 2:**
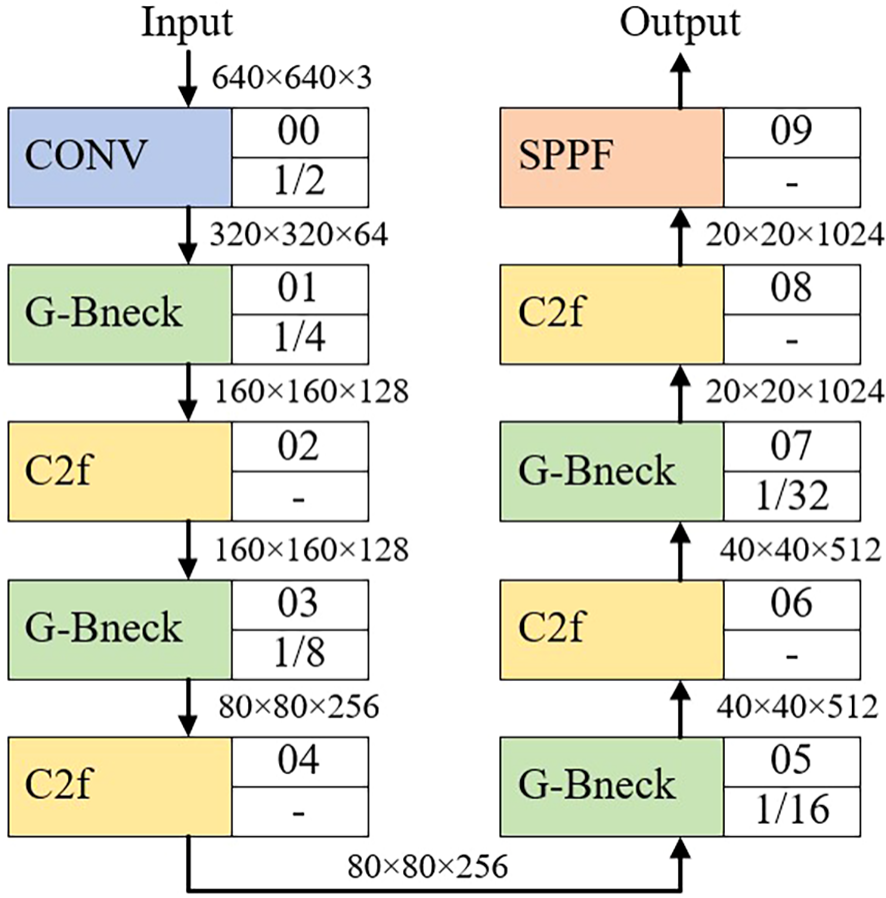
The backbone of the model after G-bneck was introduced.

The ConvModule (Han et al., 2024) in the backbone network is replaced with G-bneck, and the backbone part of the improved model is shown in **Figure 2**.

**Figure 3:**
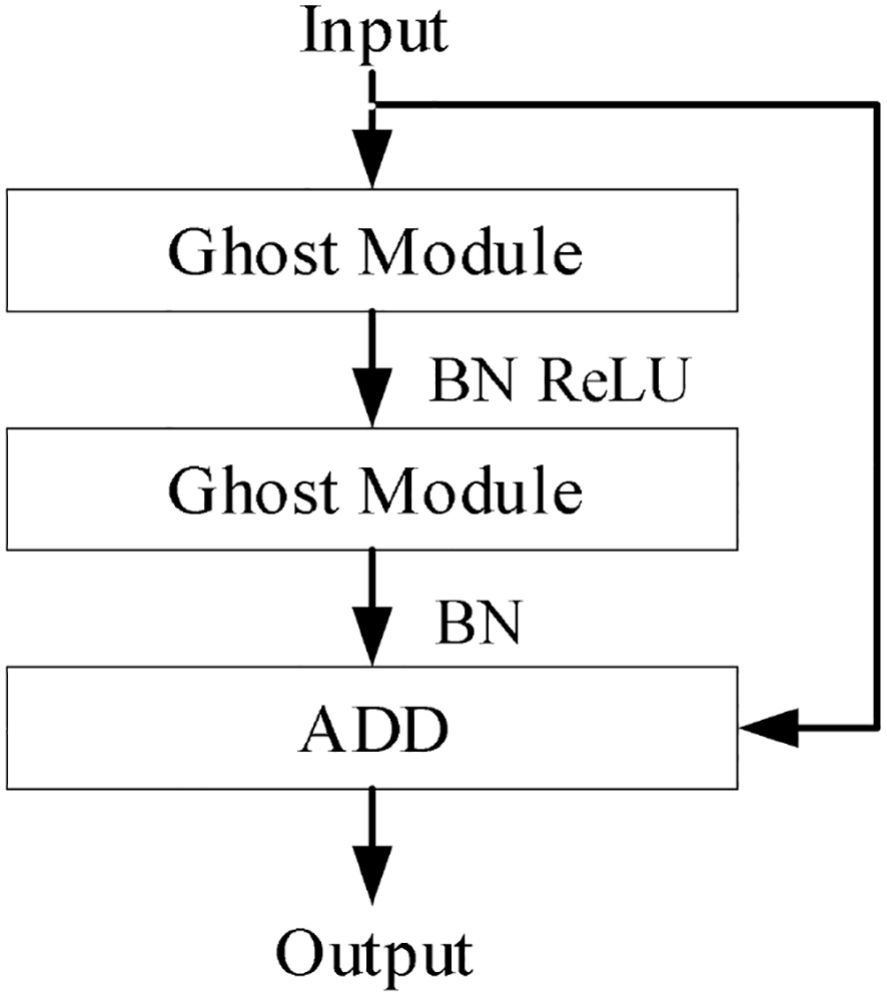
G-bneck (stride=1).

**Figure 4:**
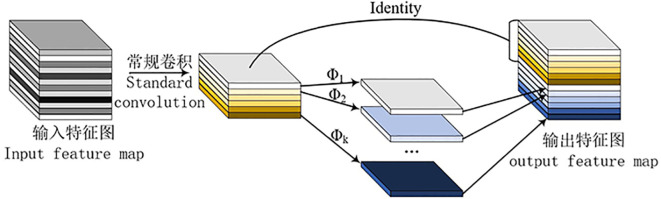
G-bneck (stride=1).

Based on the two-layer lightweight neural network basic unit GhostModule (Han et al., 2020), the ReLU activation function is added, and G-bneck is obtained after normalization. The network structure is shown in **Figure 3**. The structure of GhostModule is shown in **Figure 4**. For the input feature map, feature map A is generated through conventional convolution, then “Linear Transformation” is used to generate feature map B, and the final output feature map is obtained by concatenating (identity) feature map A and feature map B (Hu et al., 2024).

Feature map *Y"*, and each channel of the feature map ${\text{y}}_{\text{i}}^{'}$, to generate *m·(s-1)* Ghost feature maps, whose linear transformation is Equation 1:

(1) $${\text{y}}_{\text{ij}}={\text{Φ}}_{\text{i},\text{ j}}({\text{y}}_{\text{i}}^{'}),\text{∀}\text{i}=1,\ldots ,\text{m j}=1,\ldots ,\text{s}$$

Where ${\text{y}}_{\text{i}}^{'}$‘ is the i-th original feature map in *Y’*, ${\text{Φ}}_{i,j}$ is the j-th linear transformation (operation) used to generate the j-th Ghost feature map *y_ij_*, the identity mapping of the original feature map is preserved in ${\text{Φ}}_{i,j}$. The final output feature map is Equation 2:

(2) $$\text{Y}=\text{ConcatX}([{\text{Y}}^{'},{\text{Y}}^{'}\;*\;\Phi_{\text{i,j}}])$$

#### 4.1.2 Introduce the UIB module to improve the C2f module

**Figure 5:**
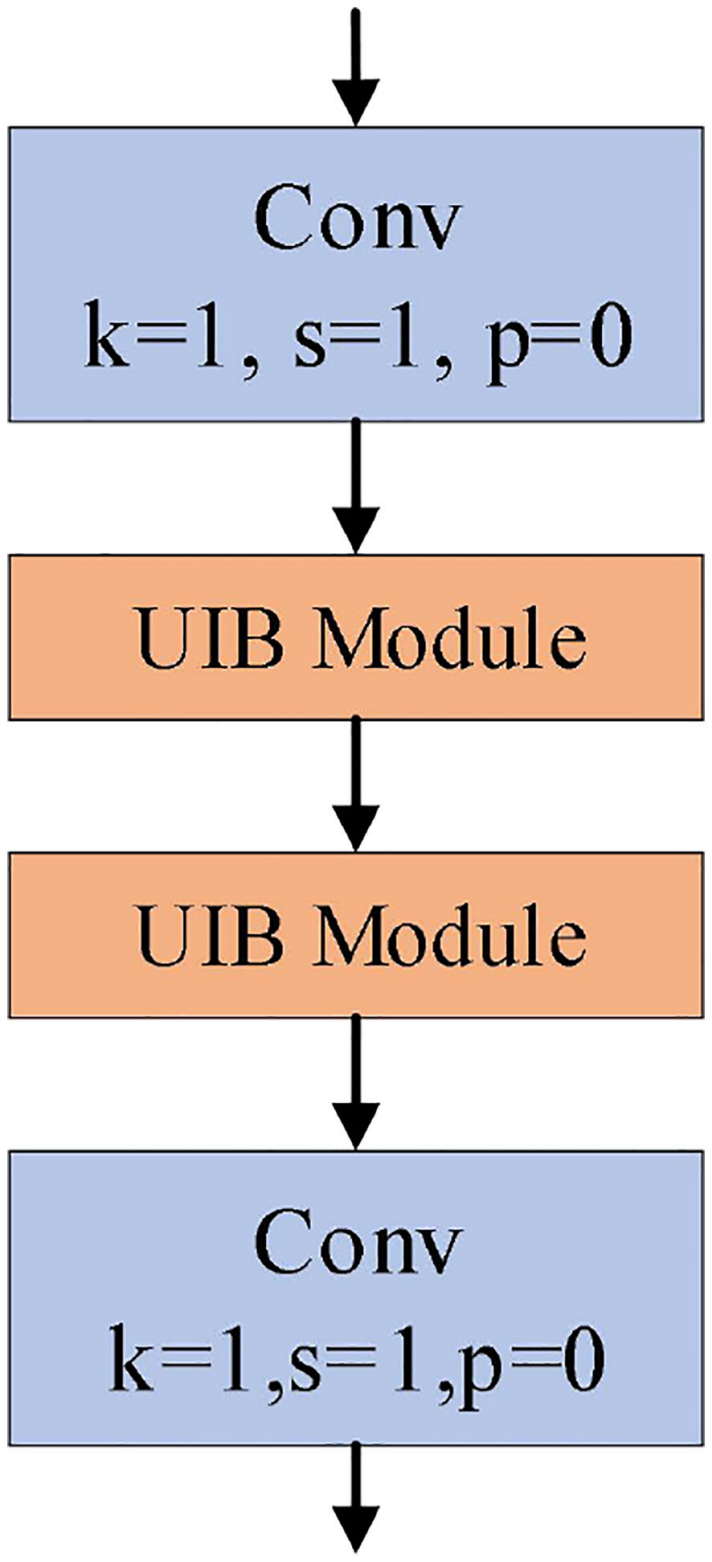
C2f-UIB module structure.

The Bottleneck module in the C2f module is replaced with the UIB module, and the skip connections are removed to obtain the structure of the C2f-UIB module, as shown in **Figure 5**.

**Figure 6:**
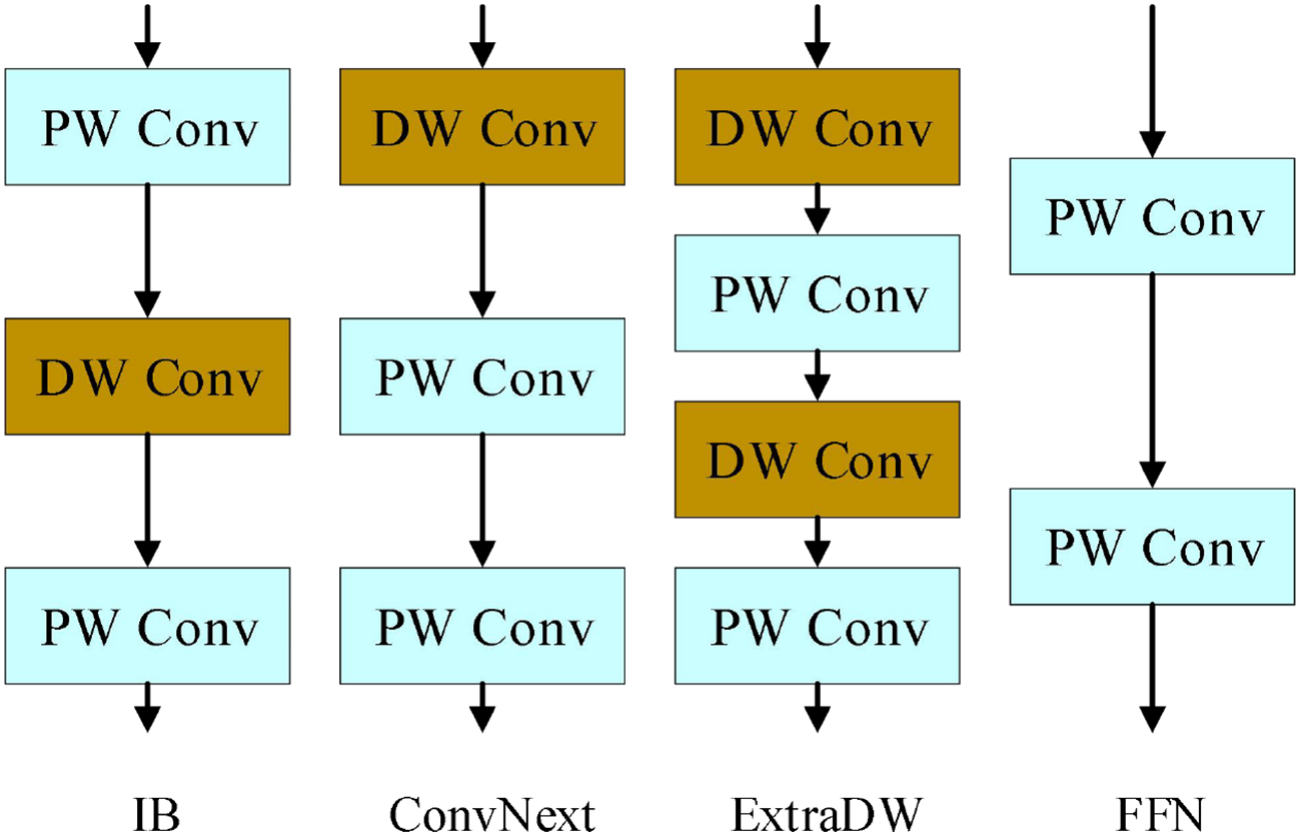
Four instantiation modes of UIB modules.

The UIB module introduces two optional depthwise convolutions into the traditional inverted bottleneck block, which are respectively located before the expansion layer and between the expansion and projection layers. The inverted bottleneck module is improved by incorporating these two depthwise convolutions, forming four possible instantiations of the UIB module. As shown in **Figure 6**.

#### 4.1.3 Replace SPPF with simSPPF

**Figure 7:**
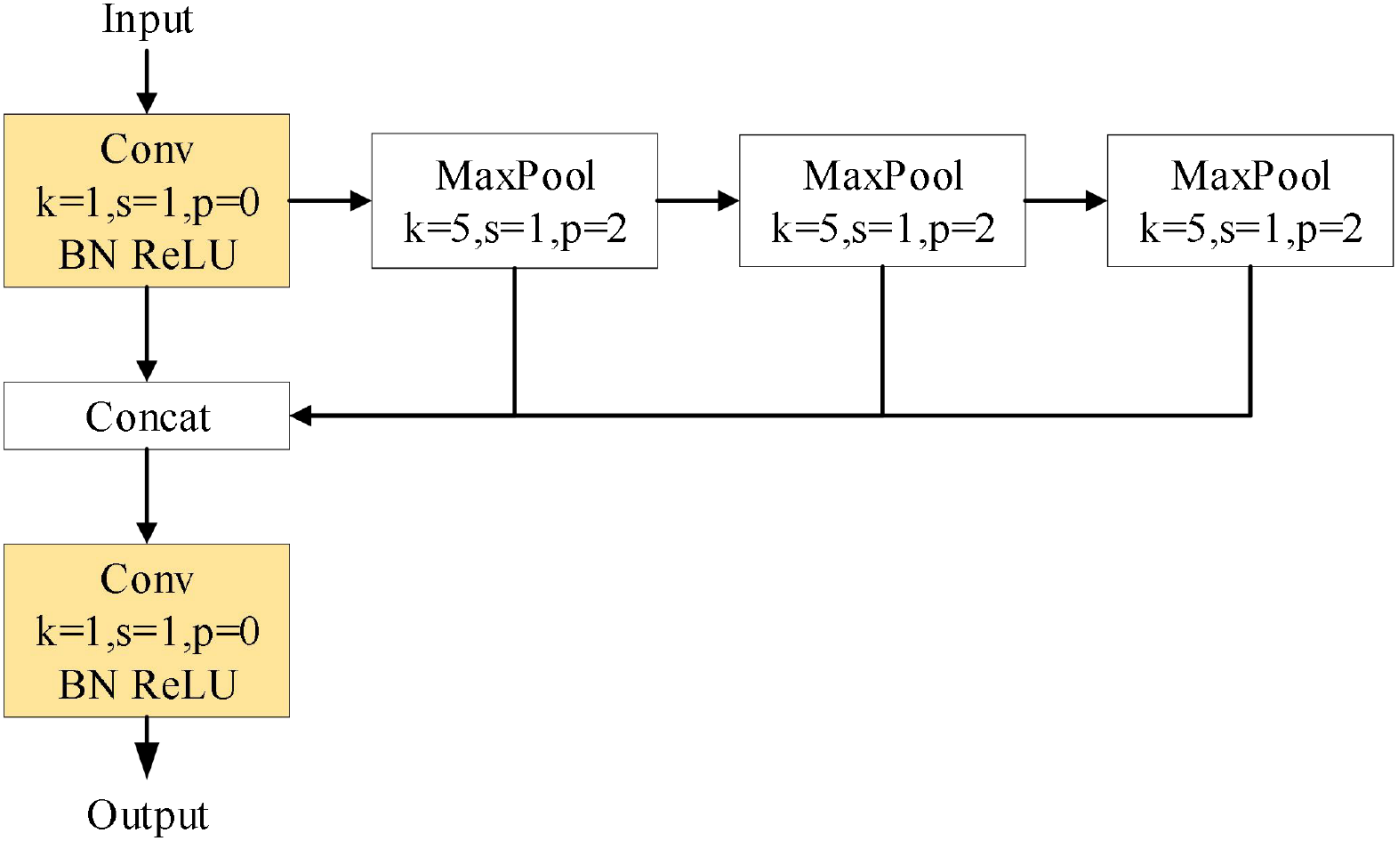
Sim-SPPF structural diagram.

SPPF improves the original SPP parallel computation into serial operation, and then replaces the SiLU activation function with the ReLU activation function to obtain simSPPF. Its structure is shown in **Figure 7**.

#### 4.1.4 Network detection head design

**Figure 8:**
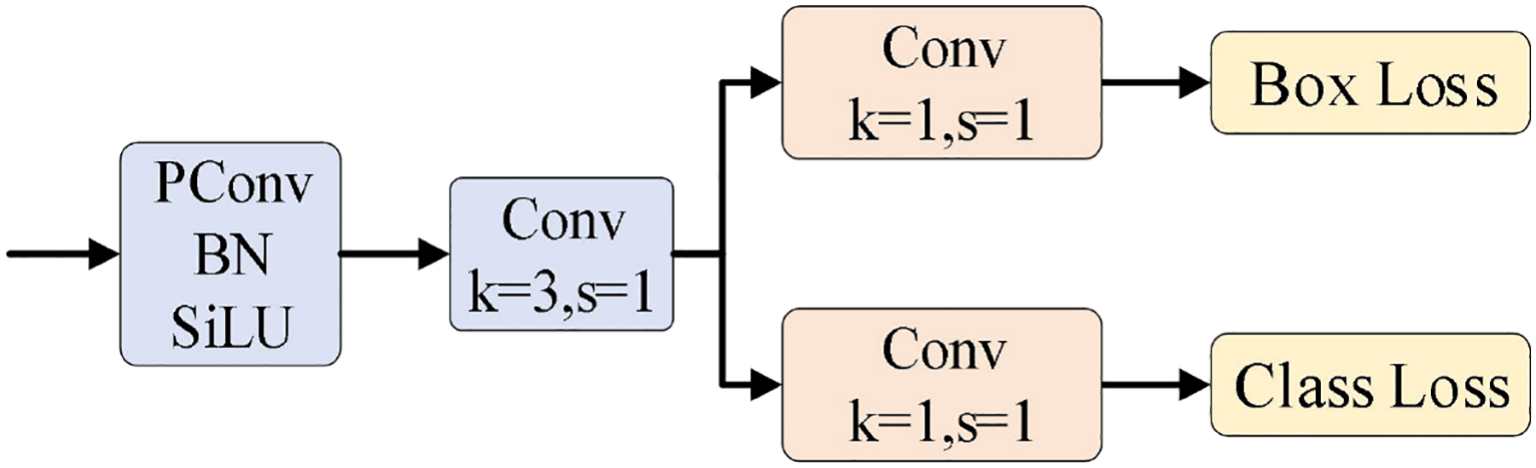
Network detection header structure.

The detection head L_Detect is obtained by introducing partial convolution (PConv) to replace the 3×3 convolution and sharing convolution parameters, thereby realizing the design of the network detection head. Its structure is shown in **Figure 8**.

1. Introduce partial convolution (PConv) to replace 3×3 convolution

**Figure 9:**
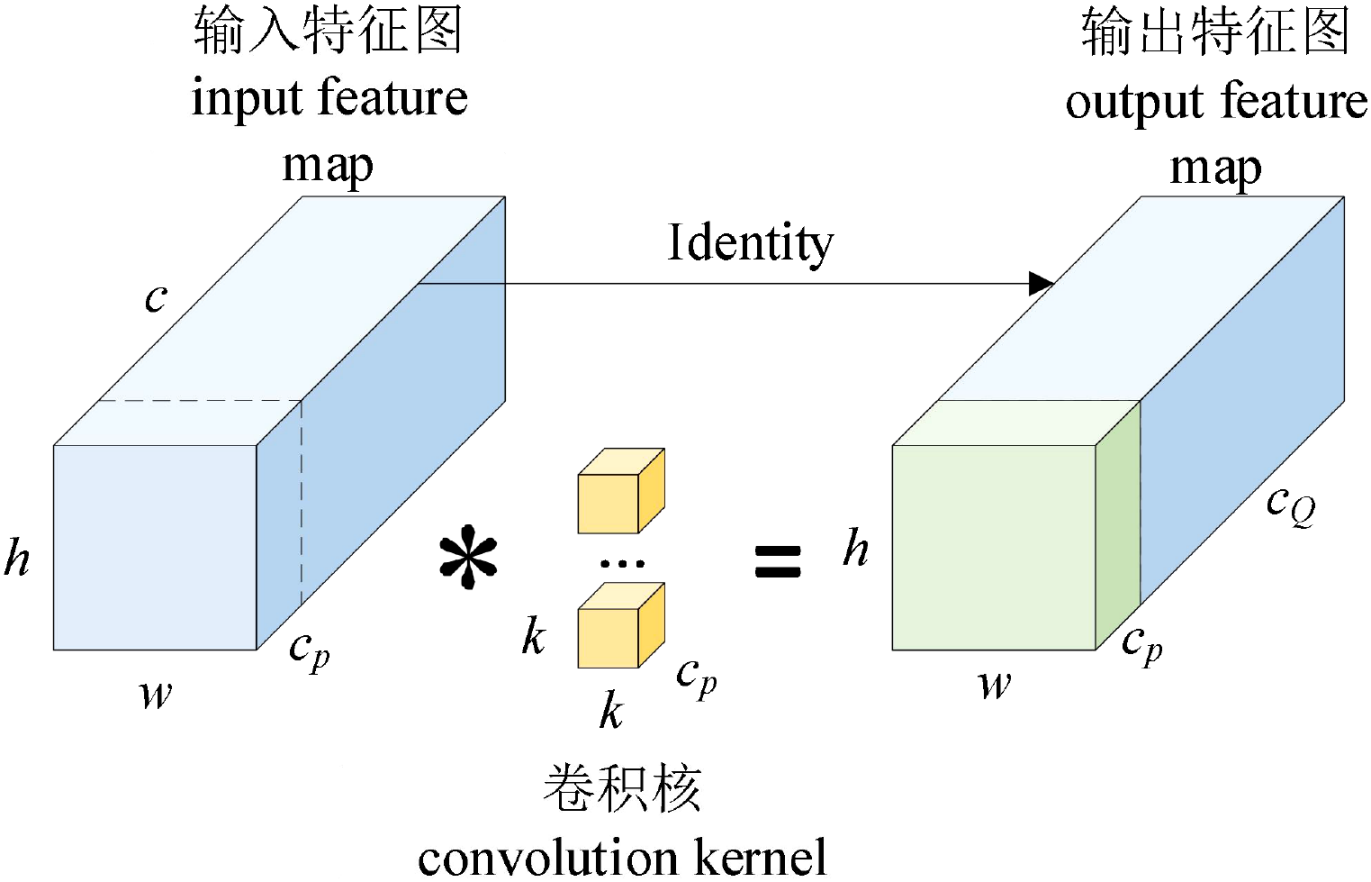
PConv schematic diagram.

Partial convolution (PConv) is introduced to replace 3×3 convolution, as shown in **Figure 9**. In PConv, some input channels undergo conventional convolution, while the remaining channels are concatenated (Identity) with the channels obtained from the conventional convolution to generate the output feature map.

2. Share convolution parameters

To accelerate the inference process of the entire model, the outputs of the same PConv and 3×3Conv are used as the inputs for the two convolution branches of the detection head, avoiding repeated computation of similar feature maps.

#### 4.1.5 Improve the C2f module and replace the loss function

The LInner-CIoU is introduced to replace the loss function LCIoU, as shown in Equation (3). Based on IoU, LInner-CIoU adds metrics for central point distance and aspect ratio, paying more attention to the alignment of central points and the consistency of dimensions. Moreover, it solves the problem of gradient vanishing in small target detection through an adjustment factor. This function focuses more on internal regional relationships and, compared with CIoU, improves training stability and detection accuracy. Applying the LInner-CIoU function to grape leaf disease detection can enhance the robustness of the model in detecting diseases under complex scenarios. The calculation of the loss function LInner-CIoU is Equation 3:

(3) $${\text{L}}_{\text{Inner}-\text{CIoU}}={\text{L}}_{\text{CIoU}}+(\text{IoU}-{\text{IoU}}^{\text{Inner}})$$

In the formula, LCIoU refers to the CIoU loss, IoU is the actual intersection over union, and IoUInner is the intersection over union between the bounding box obtained through the scaling factor and the target bounding box.

### 4.2 Experimental environment configuration and training parameter settings

The experimental operating environment is the Windows 10 operating system, with specific parameters as follows: the processor is Intel^®^ Xeon^®^ Gold 5118 Processor (16.5M Cache, 2.3GHz); 16G running memory, 4TB 7200RPM SATA hard drive; the graphics card is NVIDIA RTX2080TI-11GB; the software environment includes Anaconda4.5.11, Tensorflow2.4 framework, and cuda 11.7 computing platform.

The training parameter settings are as follows: the Adam stochastic gradient descent method is used to optimize the network model; the learning rate is set to 0.0001, the momentum parameter is set to a fixed value of 0.9, the number of iterations is epochs=300, and the number of images input per batch is batch size=16.

### 4.3 Evaluation metrics

To balance the size and speed of the grape disease recognition model, this paper evaluates the model using metrics such as Precision, Recall, mean Average Precision (mAP), Size of model, and Frames Per Second (FPS), (Luo et al., 2025). The calculation formulas are Equations 4–7:

(4) $$\text{Precision}=\frac{\text{TP}}{\text{TP}+\text{FP}}\times 100\%$$

(5) $$\text{Recall}=\frac{\text{TP}}{\text{TP}+\text{FN}}\times 100\%$$

(6) $$\text{mAP}=\frac{\sum_{\text{i}=1}^{\text{n}}\text{AP}(\text{n})}{\text{C}}\%$$

(7) $$\text{FPS}=\frac{\text{frameNum}}{\text{elapsedTime}}$$

Where TP refers to the case where the model correctly classifies positive samples as positive; FP refers to the case where the model incorrectly classifies negative samples as positive; FN refers to the case where the model incorrectly classifies positive samples as negative; TN refers to the case where the model correctly classifies negative samples as negative; n is the number of target detection categories, which is 9 in this paper; AP is the detection accuracy of a single category; C is the total number of target categories to be detected; frameNum is the total number of frames processed by the model; and elapsedTime is the total time spent by the model to process these frames.

## 5 Results and analysis

### 5.1 Comparative experiments between different models

To further verify the performance of the improved model, this paper conducts comparative experiments on YOLOv3-tiny, YOLOv5n, YOLOv6n, YOLOv7-tiny and YOLOv8 (Jocher et al., 2023) algorithms under the same conditions on a self-made dataset, using precision, recall, mean average precision (mAP), frame rate, and model size as basic evaluation metrics. The experimental results are shown in **Table 3**.

**Table 3:** Comparison results of different algorithms.

Model	Precision/%	Recall/%	Mean accuracy/%	Frame rate	Model size/MB	Training time/h
YOLOv3-tiny	91.3	84.2	83.3	129.88	23.28	43.37
YOLOv5n	89.1	87.7	89.2	156.84	5.02	39.23
YOLOv6n	90.9	88.4	86.5	182.72	8.33	33.67
YOLOv7-tiny	89.3	91.8	88.4	112.43	11.71	54.72
YOLOv8n	91.2	88.2	89.7	171.72	5.93	35.83
Improved model	94.3	87.5	92.0	228.55	3.53	26.92

According to the experimental results in **Table 3**, it can be concluded that the mean average precision (mAP) of YOLOv8n surpasses the other four networks, reaching 89.7%. Except that this model is slightly inferior to the YOLOv7-tiny model in terms of recall and slightly lower than the YOLOv6n model in terms of frame rate, its other performances are at a relatively optimal level among the five models. To sum up, YOLOv8n, as the basic network in this study, has a precision of 91.2% and a size of 5.93MB, and its comprehensive level is the best, which verifies the effectiveness of this algorithm.In terms of real-time performance, the detection speed of the improved model at 228.55 frames per second is 33% higher than that of the basic model. The training time is 26.92 hours. The size of the 3.53MB compressed model is 40% smaller than that of YOLOv8n. Under the condition of maintaining high accuracy, the computational load and the number of parameters of the model are reduced. The comprehensive performance indicators show that the improved model can better meet the requirements of high real-time performance and low resource occupation for the model during field operations while maintaining high accuracy.

### 5.2 Ablation experiments

**Figure 10:**
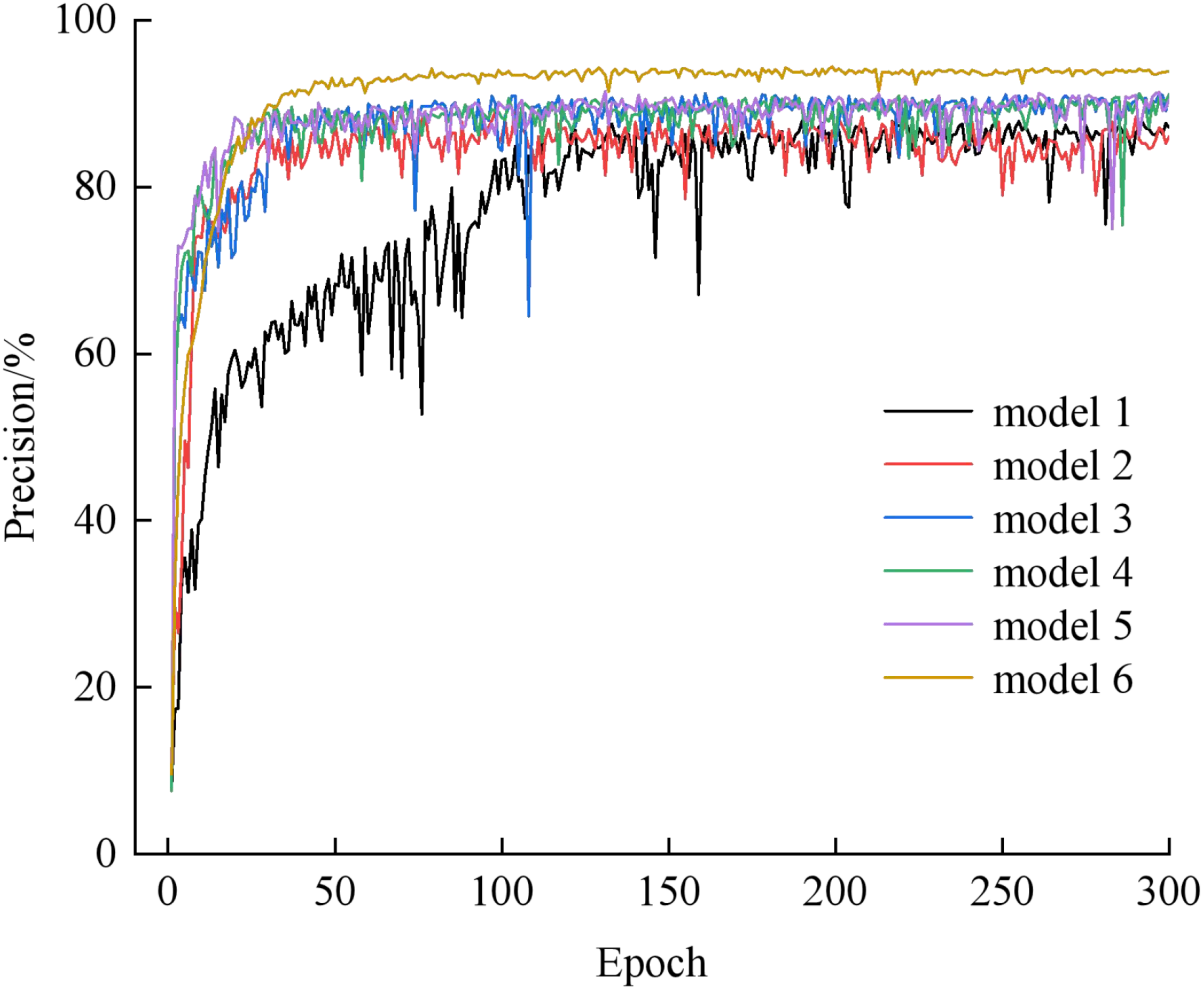
Accuracy plot.

To verify the effectiveness of the model improvements, this paper conducts ablation experiments on the YOLOv8n model. The accuracy curves of each model are shown in **Figure 10**, and the results of the ablation experiments are presented in **Table 4**, where “√” indicates the use of an improvement and “-” indicates no use of the improvement. It can be seen from **Figure 10** that by comparing the accuracy curves of the 6 models, all curves tend to converge after 300 epochs. Among them, model1 performs relatively poorly throughout the process, with low accuracy and large fluctuations. Model6 performs the best, with the highest accuracy and the most stability. The performance of other models is between model1 and model6, with relatively high accuracy but certain oscillations.

**Table 4:** Ablation test results.

Serial number	G-bneck	C2f-UIB	Improvement of the detection head	SPPF	LInner-CIoU	Precision/%	Model size/MB	Frame rate
model1	–	–	–	–	–	91.2	5.93	171.72
model 2	✓	–	–	–	–	92.3	4.60	185.12
model 3	✓	✓	–	–	–	94.1	4.24	201.34
model 4	✓	✓	✓	–	–	94.4	3.52	230.92
model 5	✓	✓	✓	✓	–	94.3	3.52	233.81
model 6	✓	✓	✓	✓	✓	97.3	3.53	228.55

It can be seen from **Table 4** that after replacing the ConvModule with the introduction of G-bneck, the model accuracy increased by 1.1%, the size decreased by 1.1MB, and the frame rate increased by 7.8%. This indicates that G-bneck generates more feature maps under the same computing resources, reduces the number of parameters and model complexity, enhances generalization ability, reduces the amount of computation, and improves processing speed. After introducing the C2f-UIB module, the model accuracy increased by 1.8%, the volume decreased by 0.36MB, and the frame rate increased by 8.8%, confirming that the UIB module is more concise and efficient compared with the original module in the C2f module. The improved detection head increased the accuracy by 0.3%, reduced the model size by 0.72MB, and increased the frame rate by 14.7%, which shows a reduction in parameters and computation. Replacing SPPF with SimSPPF decreased the accuracy by 0.3%, kept the model size unchanged, and increased the frame rate by 1.3%. Finally, after replacing the loss function with LInner-CIoU, the detection accuracy increased by 3%, the model only increased by 0.01MB, and the processing speed increased by 2.2%. To sum up, compared with the basic model, the improved YOLOv8n achieved a precision increase of 6.1 percentage points, a model size reduction of 2.40MB, and a detection speed increase of 33.90% on the self-built dataset.

### 5.3 Greenhouse experiment

**Figure 11:**
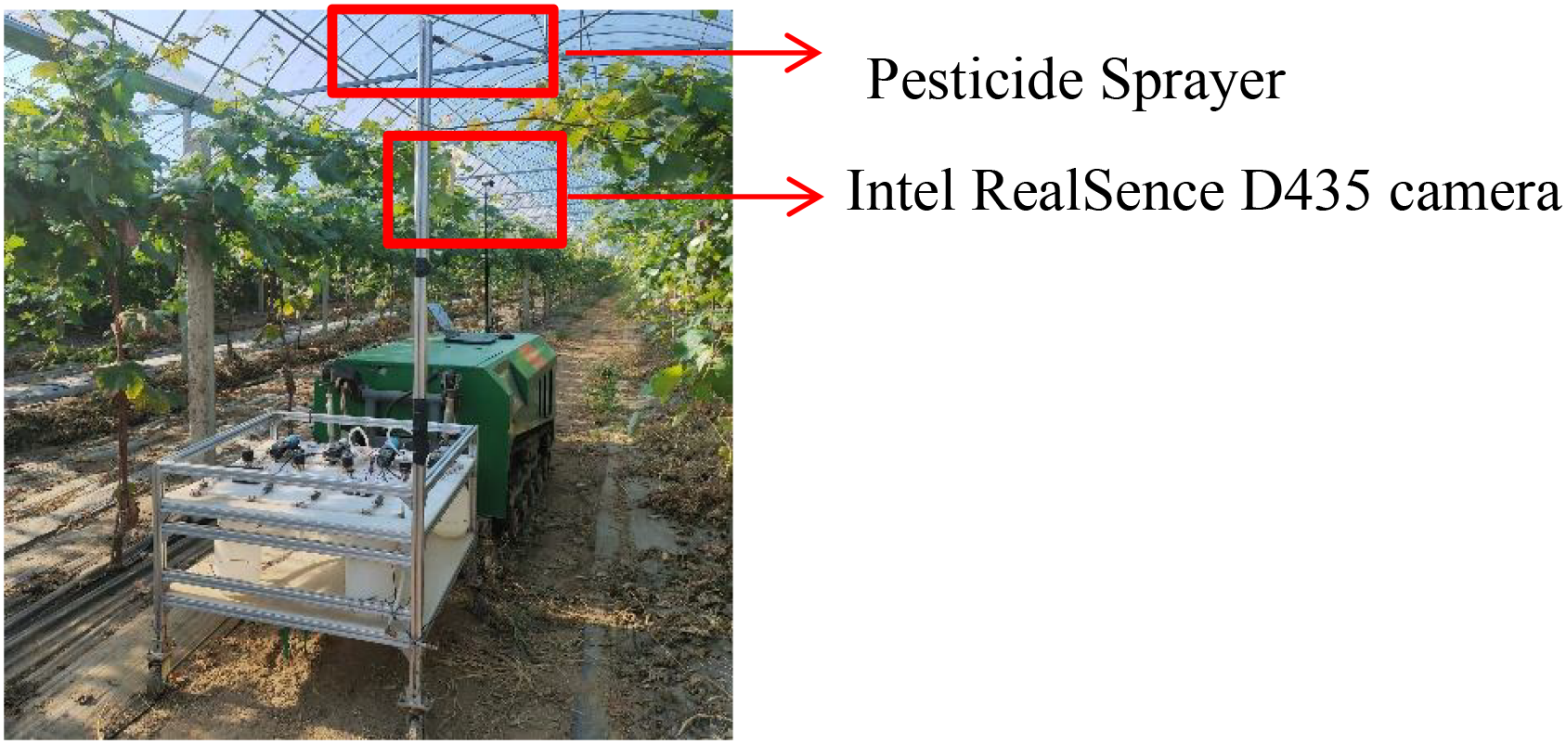
Test scenario.

To verify the recognition effect of the improved YOLOv8n grape disease recognition model in operation, the model was deployed on the grape orchard spraying device. The camera connected to the Raspberry Pi was used to obtain the disease information of fruit trees, and then the Raspberry Pi processed the images to determine the type and grade of diseases. This information was transmitted to the variable control system, which judged the type and amount of pesticide solution to be sprayed, thereby realizing variable spraying. The test scenario is shown in **Figure 11**.

The experiment was conducted in the greenhouse of the Beautiful Countryside Planting Professional Cooperative in Huyi District, Xi’an City, Shaanxi Province. After the device identified the disease images, variable spraying was carried out, and the recognition results are shown in **Table 5**. The average recognition accuracy of different disease characteristics was 89.3%, with an average time consumption of 5.18 seconds.

**Table 5:** Recognition accuracy of the YOLOv8n model at the mobile termina.

Types of diseases	Types of diseases	Number of images	Recognition results									Accuracy rate/%	Average accuracy rate/%
			H	A1	A2	B1	B2	C1	C2	D1	D2		
Healthy Leaf	H	60	54	2	1	1	0	0	0	2	0	90.0	89.3
Powdery Mildew Level 1	AI	60	3	50	3	1	2	0	1	0	0	83.3	
Powdery Mildew Level 2	AII	60	1	4	48	3	2	1	0	1	0	80.0	
Mosaic Virus Disease Level I	BI	60	2	2	4	48	2	1	0	1	0	80.0	
Mosaic Virus Disease Level II	BII	60	0	1	1	3	52	1	1	1	0	86.7	
Downy Mildew Level I	CI	60	0	0	0	2	1	53	2	1	1	88.3	
Downy Mildew Level 2	CII	60	0	0	1	0	1	3	52	2	1	86.7	
Leaf blight Level I	DI	60	0	0	0	0	0	1	1	56	2	93.3	
Leaf blight Level II	DII	60	0	0	0	0	0	0	2	3	55	91.7	

## 6 Discussion

This study took downy mildew (*Plasmopara viticola*), powdery mildew (*Erysiphe necator*), leaf blight (*Phomopsis viticola*) and Mosaic virus disease (*Grapevine mosaic virus*) of grape leaves as the research objects. The disease identification model constructed based on the improved YOLOv8n has a good effect in disease detection and identification. As these four diseases are highly prevalent and representative in the main grape-growing areas, they were selected as the research objects. To enhance the applicability of the model, although downy mildew and powdery mildew occur under different conditions in this study, we also included these two leaf diseases as part of the dataset. This data analysis model can not only be used in the spraying device of this study, but also be applied to the management links of the vineyard such as inspection, marking and cleaning of diseased plants, providing data support for subsequent decision-making.

## 7 Conclusion

To achieve rapid identification of grape diseases in orchard operating environments, this paper proposes a grape disease recognition model based on improved YOLOv8n and deploys the model on spraying equipment. The main conclusions are as follows:

1. This paper makes improvements on the basis of the YOLOv8n model. In the backbone network, G-bneck is introduced to replace ConvModule, and SimSPPF is used to replace the model’s SPPF; the optimized C2f-UIB module is introduced in both the backbone network and the neck network; in the head network, LInner-CIoU is introduced to replace the loss function LCIoU, and at the same time, partial convolution and convolution parameter sharing mechanisms are introduced in the detection head.
2. A comparative experiment was conducted between the original YOLOv8n model and YOLOv3-tiny, YOLOv5n, YOLOv6n, and YOLOv7-tiny models under the same conditions. The results show that the mean average precision of YOLOv8n surpasses the other four networks, with a precision of 91.2% and a size of 5.93MB, achieving the optimal comprehensive performance.
3. After improving the basic model, the test results show that the accuracy of the improved model reaches 97.3%, and the model size is 3.53MB. When the improved model is deployed to the spraying device, the average recognition accuracy of different disease characteristics is 89.3%, with an average time consumption of 5.18s.

This paper only targets four common types of grape disease leaves and healthy leaves. In practical applications, there are more types of crop diseases and more detailed divisions of disease severity. In future research, we will expand the types of diseases, refine the disease severity, and optimize and improve the model through a large number of experiments to enhance the applicability of the model.

## Data Availability

The raw data supporting the conclusions of this article will be made available by the authors, without undue reservation.
